# Labour classified by cervical dilatation & fetal membrane rupture demonstrates differential impact on RNA-seq data for human myometrium tissues

**DOI:** 10.1371/journal.pone.0260119

**Published:** 2021-11-19

**Authors:** Pei F. Lai, Kaiyu Lei, Xiaoyu Zhan, Gavin Sooranna, Jonathan K. H. Li, Ektoras X. Georgiou, Ananya Das, Natasha Singh, Qiye Li, Zachary Stanfield, Guojie Zhang, Rachel M. Tribe, Sam Mesiano, Mark R. Johnson

**Affiliations:** 1 Department of Metabolism, Digestion & Reproduction, Faculty of Medicine, Imperial College London, London, United Kingdom; 2 College of Life Sciences, University of Chinese Academy of Sciences, Beijing, China; 3 State Key Laboratory of Genetic Resources and Evolution, Kunming Institute of Zoology, Chinese Academy of Sciences, Kunming, China; 4 Systems Biology and Bioinformatics Program, Case Western Reserve University, Cleveland, Ohio, United States of America; 5 Department of Biology, Section for Ecology and Evolution, University of Copenhagen, Copenhagen, Denmark; 6 Department of Women and Children’s Health, School of Life Course Sciences, King’s College London, London, United Kingdom; 7 Department of Reproductive Biology, Case Western Reserve University, Cleveland, Ohio, United States of America; 8 Department of Obstetrics and Gynecology, University Hospitals of Cleveland, Cleveland, Ohio, United States of America; Azienda Ospedaliero Universitaria Ospedali Riuniti di Ancona Umberto I G M Lancisi G Salesi, ITALY

## Abstract

High throughput sequencing has previously identified differentially expressed genes (DEGs) and enriched signalling networks in human myometrium for term (≥37 weeks) gestation labour, when defined as a singular state of activity at comparison to the non-labouring state. However, transcriptome changes that occur during transition from early to established labour (defined as ≤3 and >3 cm cervical dilatation, respectively) and potentially altered by fetal membrane rupture (ROM), when adapting from onset to completion of childbirth, remained to be defined. In the present study, we assessed whether differences for these two clinically observable factors of labour are associated with different myometrial transcriptome profiles. Analysis of our tissue (‘bulk’) RNA-seq data (NCBI Gene Expression Omnibus: GSE80172) with classification of labour into four groups, each compared to the same non-labour group, identified more DEGs for early than established labour; ROM was the strongest up-regulator of DEGs. We propose that lower DEGs frequency for early labour and/or ROM negative myometrium was attributed to bulk RNA-seq limitations associated with tissue heterogeneity, as well as the possibility that processes other than gene transcription are of more importance at labour onset. Integrative analysis with future data from additional samples, which have at least equivalent refined clinical classification for labour status, and alternative omics approaches will help to explain what truly contributes to transcriptomic changes that are critical for labour onset. Lastly, we identified five DEGs common to all labour groupings; two of which (*AREG* and *PER3*) were validated by qPCR and not differentially expressed in placenta and choriodecidua.

## Introduction

Understanding how parturition (i.e. the process of birth) is initiated, specifically how the myometrium (uterine smooth muscle) is activated to generate the contractions of labour, is essential if we are to reduce rates of adverse maternal and fetal/neonatal outcomes associated with aberrant timing of birth, such as preterm birth and prolonged pregnancy. Preterm birth (i.e. birth prior to 37 weeks of gestation) affects 10–15% of pregnancies worldwide and is the leading cause of death for children under the age of 5 years (including neonates and infants) [1]. The majority (~70%) of preterm births occur spontaneously without clear etiology or cause [2]. Prolonged pregnancy (>41 weeks of gestation; late and post term) is also problematic as it increases the risk of stillbirth and significant neonatal morbidity [3]. Current clinical strategies to prevent preterm labour, stop preterm labour after it has started, or induce labour at late/post term pregnancy are relatively unsuccessful for improving maternal and neonatal outcomes [4, 5]; this is mostly due to our incomplete understanding of the physiological process of term labour and the pathological mechanisms that result in its mistiming.

Human labour can be clinically divided into two distinct phases: (i) ‘early’ phase, which is characterised by cervical effacement, with increasing frequency/intensity of uterine contractions and ≤3 cm cervical dilatation, and (ii) ‘established’ phase, which is characterised by regular, strong uterine contractions and >3 cm cervical dilatation. Rupture of fetal (amniotic and chorionic) membranes (ROM), which decreases intrauterine pressure with release of (cytokines-containing) amniotic fluids, occurs after contractions have initiated for most pregnancies [6]. Transcriptional changes responsible for initiating uterine contractions to start parturition are more likely to be detected in myometrium samples obtained during early labour, whereas consequential changes are expected to dominate observations for established labour; whether ROM can alter the myometrial transcriptome irrespective of labour status defined by cervical dilatation has not been previously determined. In fact, most published myometrium transcriptome studies [7–17] have compared samples without clear differential analysis for these factors of labour and, in some cases, samples were obtained from women after clinical interventions to artificially augment the process; both shortfalls have potential to obscure the identities of true (i.e. endogenous) labour initiators [18, 19].

Previously, we used whole tissue (‘bulk’) RNA-seq to obtain a transcriptomics dataset for myometrial biopsies from singleton pregnant women at term gestation, who were clinically classified as (i) not in labour (TNL), (ii) in early phase labour (TEaL) or (iii) in established phase labour (TEsL), which has already been used for an integrated analysis with those from two other myometrium-based studies (one also RNA-seq [20] and another that used microarrays [15]). Although this identified parturition-related signalling networks from a combined total of 33 non-labouring and 38 labouring women [21], the data from our TEaL and TEsL samples were combined as one labour group so that the impact of cervical dilatation, as well as ROM, were not differentiated. Thus, it remained unclear which genes and signalling networks were most likely to be responsible for starting the process of labour.

In the present study, we applied a different bioinformatics approach to only our dataset to assess transcriptomic changes that occur in the myometrium at different stages of physiological labour. We specifically investigated whether refining classification of term labour by cervical dilatation or ROM status would result in different profiles of DEGs and enriched gene ontology (GO) terms. Our observations highlight factors of myometrial tissue heterogeneity, which will be discussed for the intention of improving design of future omics-based studies. Additionally, we used a new (‘second’) sample cohort to validate five DEGs, which were identified from our RNA-seq cohort during the present study to be common to all our labour classifications of interest. We also determined to what extent these labour-associated DEGs of interest were distinct to myometrium, when compared to their expression patterns in cohort-matched placenta and choriodecidua.

## Materials and methods

### Ethical approval

Myometrium, placenta and choriodecidua biopsies from women undergoing Caesarean section with singleton pregnancies at term (37^+3^–41^+1^ weeks) gestation were obtained with written consent in accordance with the Declaration of Helsinki guidelines, and with approval from the Brompton and Harefield Research Ethics Committee (London, UK; Ethics No. 10/H0801/45).

### Study setting and participant selection

#### Setting

The study was carried out in Chelsea & Westminster Hospital NHS Foundation Trust, a tertiary referral teaching hospital in London, England, UK.

#### Participants

All women in the study underwent Caesarean section following medical advice provided by their clinical care team at Chelsea & Westminster Hospital. Following this, recruitment to the study and related documentation of clinical data were conducted by obstetricians (clinical research fellows and consultants) and a postdoctoral scientist within the research term.

#### Subject categorisation

Participants were firstly categorised as TNL, TEaL or TEsL, as described previously for the RNA-seq cohort [21], for all 38 women in the present study. Briefly, labour was defined by the presence of both regular palpable uterine contractions (≥1–2 per 10 minutes; assessed using cardiotocography) and progressive cervical dilatation (assessed using digital examination) [22]; TNL women presented with no palpable uterine contractions and a closed cervix. Labouring women were further categorised as either TEaL (≤3 cm dilated at cervix; both cohorts) or TEsL (>3 cm dilated at cervix; RNA-seq cohort only) immediately prior to Caesarean section. Additionally, ROM status of labouring women was determined during speculum examination of the vaginal cavity prior to Caesarean section. For the present study, women were categorised as labouring without ROM (TL-ROM) if ROM was documented as present for ≤1 hour (i.e. ROM negative or ROM occurred in the operating theatre), or labouring in the presence of ROM (TL+ROM) if ROM occurred >1 hour, prior to fetal delivery.

#### Exclusion criteria

For both cohorts, women with diabetes (gestational, type I and type II), preeclampsia, obstetric cholestasis, signs of an infection, who were administered drugs for labour induction or augmentation (i.e. prostaglandins and oxytocin), or who were having Caesarean section for failure to progress were excluded.

#### Study duration

Time taken to obtain all samples for the RNA-seq (n = 22) and second (n = 16) cohort was 17 and 6 months, respectively.

### Tissue samples collection

Myometrium biopsies from the upper edge of Caesarean section incision, made to the lower uterine segment, were excised prior to completion of suturing after fetal and placental delivery; all participating pregnancies resulted in live births. Biopsies of placenta (maternal side; region adjacent to umbilical cord insertion) and chorionic membrane (after manual separation from amniotic membrane and without removing decidua; i.e. choriodecidua) were collected after their routine clinical checks were completed outside of the operating theatre. Myometrium and placenta biopsies were immediately washed with ice-cold sterile Dulbecco’s phosphate-buffered saline (Sigma-Aldrich, Dorset, UK), dissected into ~3 mm^3^ pieces, treated with RNAlater (Sigma-Aldrich) overnight at 2–4°C and transferred into -80°C storage prior to RNA extraction, as described (for myometrium) previously [21]; the same process was undertaken for choriodecidua except dissected tissues were flash frozen in liquid nitrogen instead of using RNAlater.

### RNA extraction, library preparation, sequencing and data processing for RNA-seq

RNAlater-treated myometrial tissues were used for RNA extraction as described previously [21], along with RNA quality assessment, preparation of cDNA libraries and strand-specific RNA-seq; the latter utilised a HiSeq 2000 instrument (Illumina, San Diego, USA) to generate an average of 42 million DNA fragments per sample (100 base pair paired-end reads; strand-specific) and FastQC software (version 0.11.2; Babraham Institute, Cambridge, UK) was used for quality control, all undertaken at the Imperial BRC Genomics Facility (Imperial College London, UK). The raw dataset has been deposited into NCBI’s Gene Expression Omnibus (GEO) with series accession number GSE80172 [21].

RNA-seq reads were aligned to the GRCh38 *Homo sapiens* reference genome provided by the Ensembl project (release 84) [23] using HISAT2 (version 2.1.0) [24, 25] with parameters of–dta-cufflinks–fr–phred33 –p 4 –q. Index was built with the information about single nucleotide polymorphisms (SNPs) and annotated transcripts. Ensembl annotated a total of 58825 genes, which included 20465 protein-coding genes. A transcript merging procedure was implemented to produce gene level models for expression analysis. Specifically, exons labelled as ‘retained_intron’ were first excluded, then overlapping interval exons of each gene were merged and a final gene level model was produced in general feature format (GFF). Only uniquely mapped reads, where a NH:i:1 tag was present, were used to produce gene read counts. An average of 53 million reads were obtained from each sample. More than 94.92% of total reads were successfully aligned to the GRCh38 reference human genome and unique concordant pair ratio was greater than 88.57%. In total, 37082 genes were mapped with the following criteria: (i) at least one RNA-seq read was assigned to a gene, and (ii) a read was only assigned to a gene when >90% of this read matched the exon regions of the gene. A gene expression matrix and design of the experiment were provided to DESeqDataSetFromMatrix function from DESeq2 (version 1.6.3) [26]; expression values presented in transcripts per million (TPM) units at S1 and S2 Datasets.

Feature normalization was conducted by rlog function to transform the matrix to log2 scale and principal component analysis (PCA) was performed by principal function to produce the top ten principal components. DEGs between sample groups were identified using DESeq2, edgeR (version 3.8.6) [27] and baySeq (version 2.4.1) [28] differential expression analysis packages. Raw *p* values were adjusted by false discovery rate (FDR) to produce *q* values (i.e *p* values corrected to account for multiple comparisons between sample groups); a *q* value of 0.05 was chosen as the cut-off for statistical significance in DESeq2, edgeR and baySeq. Fold change (FC) ≥1.5 in median TPM, whereby the larger TPM was always divided by the smaller TPM, between two sample groups of interest were used for GO enrichment and Venn diagrams; the expression FC was calculated as a ratio of median, rather than mean, TPM to minimise impact from large variance between samples.

Enrichment Analysis for Customised Organism (EACO) package [29] was used for its computational pipeline to undertake statistical analysis of GO enrichment; genes with a median TPM≥1.5 in at least one of the two sample groups of paired comparison were used as background. Fisher’s exact test was used to identify whether DEGs (foreground genes) were enriched in a specific GO category when compared to background genes; *p* values were adjusted for multiple testing using the Benjamini-Hochberg procedure for FDR [30]. Venn diagrams were drawn using the jvenn online tool [31] to visualise FC≥1.5 DEGs lists from all pairs of comparisons made to TNL; their accompanying gene lists were interpreted using the Human Genome Organisation (HUGO) Gene Nomenclature Committee (HGNC) Gene BioMart online tool [32].

### qPCR

Total mRNA was extracted and purified from all tissues using the TRIzol Plus RNA Purification kit (Life Technologies, Paisley, UK). After Nanodrop quantification, 1.0 μg RNA was reverse transcribed using the QuantiTect Reverse Transcription kit (Qiagen, Manchester, UK). SYBR Green (Life Technologies) was used for qPCR with a Rotor-Gene Q thermocycler (Qiagen); DNA denaturation, annealing and extension steps were as described previously [33], and qPCR standards (prepared from pooled second cohort cDNA samples) were defined by copy number. Nucleotide sequences for qPCR primers are listed in Table 1, which were designed using NCBI Primer-BLAST [34] and purchased from Life Technologies. Data for cohort-matched samples were acquired during the same set of qPCR cycles for each pair of primers so that relative expression patterns were comparable between tissue types. Each DEG was normalised to two housekeeping genes, β2-microglobulin (*B2M*) and ribosomal protein L19 (*RPL19*); the geometric mean of these normalised copy numbers was calculated for each sample and all subsequently log_10_ transformed for data presentation.

**Table 1:** Primers for qPCR.

Name	Forward (F) and Reverse (R) Primer Sequence (5’ to 3’)	RefSeq Accession Number	PCR Product Size (bp)
***AREG***	F: tgtcgctcttgatactcggc	NM_001657	173
	R: aggcatttcactcacagggg		
***LIF***	F: gccacccatgtcacaacaac	NM_002309	140
	R: gccacatagcttgtccaggt		
***LILRA5*** ^a^	F: cacgtgcaggcagggaa	NM_021250; NM_181879	159
	R: ctgtgtgtcccagggttctg		
***NAMPT***	F: ggagcatctgctcacttggt	NM_005746	155
	R: tcatggtctttcccccaagc		
***PER3***	F: atggcagtgagagcagtcct	NM_001289862; NM_001289861; NM_001289863; NM_016831; NM_001289864; NM_001377276; NM_001377275	157 / 211
	R: aatcccatggacagtgtgct		
***B2M***	F: tgggtttcatccatccgaca	NM_004048	160
	R: acggcaggcatactcatctt		
***RPL19***	F: caggcacatgggcataggtaa	NM_000981; NM_001330200	165
	R: ttcaccttcaggtacaggct		

^***a***^ *LILRA5* can be expressed as four different isoforms; we have presented data using primers designed for the mRNA sequences of isoforms 1 & 3 (also known as LIR9m1 & LIR9s1) because our primers for isoforms 2 & 4 (LIR9m2 & LIR9s2) produced poor qPCR product yield; we were unable to design primers that could detect mRNA sequences that were common to all four *LILRA5* isoforms.

### Statistical analyses

Prism 8.0 (GraphPad, San Diego, USA) was used for statistical analyses of patient demographics and qPCR data; their fit to normal distribution was assessed using the Shapiro-Wilk test. For patient demographics, data were analysed using non-parametric two-tailed Mann-Whitney or Kruskal-Wallis (Dunn’s *post hoc*) test. For qPCR data, all geometric mean values were log_10_ transformed to permit parametric analysis (of lognormal populations) using a two-tailed Welch’s *t* test or Brown-Forsythe & Welch (Dunnett’s T3 *post hoc*) ANOVA. Statistical significance was identified as *p*≤0.05 and *p* values from comparisons between ≥3 groups were adjusted for multiplicity of pairings.

## Results

### Participant demographics and clinical characteristics

Demographic and clinical characteristics details for all participants are summarised in Tables 2 and 3 (RNA-seq cohort [21]; n = 22) and Table 4 (second cohort; n = 16), where reasons for Caesarean section are presented.

**Table 2:** Patient demographics for myometrium & placenta biopsies of the RNA-seq cohort—Grouped by phases of labour as determined by cervical dilatation.

	Term No Labour (TNL, No Cervical Dilation; n = 8)	Term Early Labour (TEaL, ≤3 cm Cervical Dilation; n = 8)	Term Established Labour (TEsL, >3 cm Cervical Dilation; n = 6)	*p* Values from Kruskal-Wallis Test (All Groups)
***Maternal Age (Years; Median & Range)***	34.0 (27–39)	35.5 (29–38)	32.5 (29–37)	0.529
***Gestational Age (Weeks* ^*+Days*^ *; Median & Range***	39^+0^ (38^+5^–40^+0^)	40^+0^ (37^+3^–40^+5^)	39^+0^ (38^+3^–41^+1^)	0.866
***Gravida (Median & Range)***	1 (1–4)	2 (1–4)	2 (1–6)	0.671
***Parity (Median & Range)***	Viable = 0 (0 to 1) Non-viable & abortus = 0 (0 to 3)	Viable = 1 (0 to 2) Non-viable & abortus = 0 (0 to 2)	Viable = 1 (0 to 1) Non-viable & abortus = 0 (0 to 3)	0.596 0.770
***Booking Body Mass Index (BMI*, *kg/m*^*2*^*; Median & Range)***	21 (19 to 25)	22 (19 to 24)	20 (19 to 21)	0.051 (Dunn’s *post hoc*: TEsL *vs* TEaL = 0.050)
***Ethnicity (as self-specified by participant)***	White European = 3 British White other = 1 North American (USA) Stated as ‘other’ = 1 unspecified Not stated = 3	White European = 4 British, 1 Russian White other = 1 Brazilian Mixed ethnicity = 1 unspecified Not stated = 1	White European = 3 White British, 1 unspecified White other = 1 South African Mixed ethnicity = 1 White & Black Caribbean	n/a
***Reason for Caesarean Section***	Breech = 3 Maternal request: Personal choice = 1; Previous traumatic delivery = 2; Tocophobia = 2	Breech = 3 Fetal distress = 1 Maternal request: Previous Caesarean = 3; Previous traumatic delivery = 1	Breech = 4 Fetal distress = 1 Maternal request: Previous traumatic delivery = 1	n/a
***Rupture of Fetal Membranes***	None	Spontaneous = 2; Artificial = 1	Spontaneous = 4; Artificial = 1	n/a
***Time of Birth by Caesarean Section (hh*:*mm*, *24 h clock format)***	09:46, 09:56, 10:07, 10:54, 11:58, 12:25, 13:23, 13:32	01:45, 06:57, 15:02, 15:10, 18:33, 20:02, 20:12, 22:08	06:16, 07:28, 10:19, 11:00, 12:26, 23:25	0.306

**Table 3:** Patient demographics for myometrium & placenta biopsies of the RNA-seq cohort—Grouped by status of fetal membrane rupture (ROM).

	Term No Labour (TNL, No Cervical Dilation; n = 8)	Term Labour with Absence of ROM (TL-ROM; n = 8)	Term Labour with Presence of ROM (TL+ROM; n = 6)	*p* Values from Kruskal-Wallis Test (All Groups)
***Maternal Age (Years; Median & Range)***	34.0 (27–39)	35.0 (30–38)	32.5 (29–37)	0.552
***Gestational Age (Weeks* ^*+Days*^ *; Median & Range)***	39^+0^ (38^+5^–40^+0^)	40^+0^ (37^+3^–41^+1^)	38^+4^ (38^+3^–40^+3^)	0.260
***Gravida (Median & Range***	1 (1–4)	2 (1–6)	2 (1–4)	0.547
***Parity (Median & Range)***	Viable = 0 (0 to 1) Non-viable & abortus = 0 (0 to 3)	Viable = 1 (0 to 2) Non-viable & abortus = 0 (0 to 3)	Viable = 1 (0 to 1) Non-viable & abortus = 0 (0 to 2)	0.596 0.605
***Booking Body Mass Index (BMI*, *kg/m*^*2*^*; Median & Range)***	21 (19 to 25)	21 (19 to 24)	21 (20 to 23)	0.893
***Ethnicity (as self-specified by participant)***	White European = 3 British White other = 1 North American (USA) Stated as ‘other’ = 1 unspecified Not stated = 3	White European = 4 British, 1 Russian White other = 1 South African Mixed ethnicity = 1 White & Black Caribbean Not stated = 1	White European = 3 British, 1 unspecified White other = 1 Brazilian Mixed ethnicity = 1 unspecified	n/a
***Reason for Caesarean Section***	Breech = 3 Maternal request: Personal choice = 1; Previous traumatic delivery = 2; Tocophobia = 2	Breech = 4 Fetal distress = 1 Maternal request: Previous traumatic delivery = 1; Previous Caesarean = 2	Breech = 3 Fetal distress = 1 Maternal request: Previous traumatic delivery = 1; Previous Caesarean = 1	n/a
***Rupture of Fetal Membranes***	None	Spontaneous (<1 h) = 1 Artificial (<1 h) = 1	Spontaneous (>1 h) = 5 Artificial (>1 h) = 1	n/a
***Time of Birth by Caesarean Section (hh*:*mm*, *24 h clock format)***	09:46, 09:56, 10:07, 10:54, 11:58, 12:25, 13:23, 13:32	01:45, 06:16, 06:57, 07:28, 11:00, 15:10, 18:33, 20:02	10:19, 12:26, 15:02, 20:12, 22:08, 23:25	0.067

**Table 4:** Patient demographics for myometrium, placenta & choriodecidua biopsies used as independent ‘second cohort’ for qPCR validation of RNA-seq findings.

	Term No Labour (TNL, No Cervical Dilation; n = 8)	Term Early Labour (TEaL, ≤3 cm Cervical Dilation; n = 8)	*p* Values from Mann-Whitney Test (All Groups)
***Maternal Age (Years; Median & Range)***	34.5 (31–38)	34.0 (31–42)	0.741
***Gestational Age (Weeks* ^*+Days*^ *; Median & Range)***	39^+1^ (38^+4^–39^+5^)	38^+5^ (37^+3^–40^+0^)	0.152
***Gravida (Median & Range)***	2 (1–3)	2 (2–4)	0.094
***Parity (Median & Range)***	Viable = 1 (0–2) Non-viable & abortus = 0 (0–1)	Viable = 1 (0–2) Non-viable and abortus = 1 (0–1)	0.543 0.282
***Booking Body Mass Index (BMI*, *kg/m*^*2*^*; Median & Range)***	22.0 (18.1–38.0)	21.9 (19.9–23.4)	>0.999
***Ethnicity (as self-specified by participant)***	White European = 2 British, 1 Bulgarian, 1 Dutch, 1 Irish, 1 Lithuanian Asian = 1 Armenian Stated as ‘other’ = 1 unspecified	White European = 2 British, 1 Italian, 1 Irish Asian = 1 Indian, 1 Sri Lankan White other = 1 Australian, 1 South American (Argentina)	n/a
***Reason for Caesarean Section***	Breech / transverse lie = 3 Maternal request: Previous traumatic delivery = 3 Placenta previa = 1 Recurrent perineal abscess and fistula = 1	Breech = 2 Maternal request: Previous Caesarean = 6	n/a
***Rupture of Fetal Membranes***	None	Spontaneous = 3 Artificial = 0	n/a
***Time of Birth by Caesarean Section (hh*:*mm*, *24 h clock format)***	09:29, 09:32, 09:48, 11:15, 11:17, 11:19, 12:46, 16:39	09:13, 09:29, 10:10, 12:44, 12:57, 15:26, 15:31, 16:03	0.594

### DEGs associated with labour classified by status of cervical dilatation

**Fig 1:**
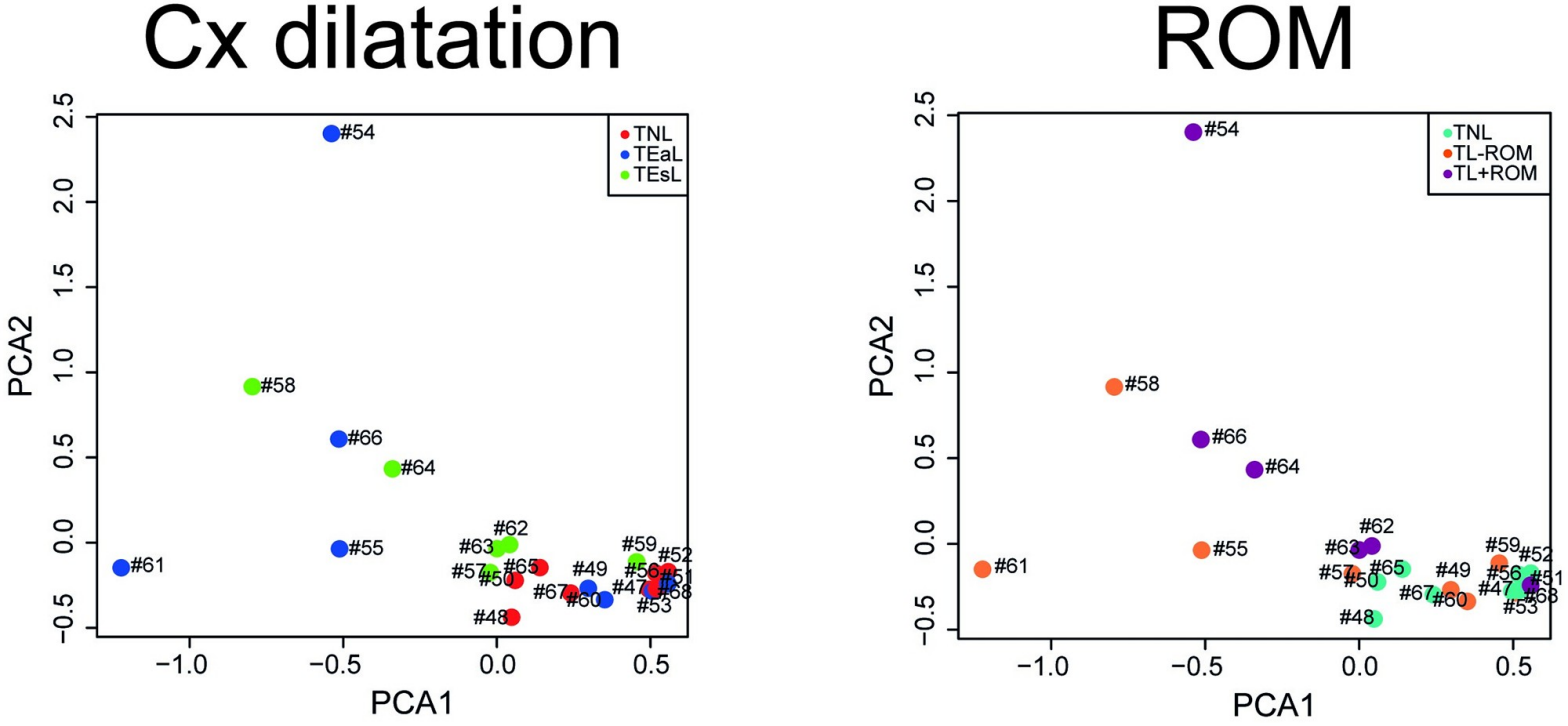
PCA for RNA-seq of myometrium biopsies from term pregnant women at different stages of labour. Summary by principal component analysis (PCA) of overall differences in transcriptome profiles from human myometrium biopsies obtained from term gestation singleton pregnant women, which were grouped by status of cervical (Cx) dilatation or fetal membrane rupture (ROM; >1 hour prior to fetal delivery) at time of Caesarean section; sample identification numbers are shown next to their respective data points. For Cx dilatation, labouring women were grouped as either in early (≤3 cm; TEaL, n = 8) or established (>3 cm; TEsL, n = 6) labour. For ROM, women were grouped as either labouring in the absence (TL-ROM, n = 8) or presence (TL+ROM, n = 6) of ROM. The non-labouring (TNL, n = 8) group was the same for both sets of comparisons.

To evaluate the overall impact of cervical dilatation (Table 2) on myometrial transcriptome profiles, samples from women within the RNA-seq cohort were grouped as TNL (n = 8), TEaL (n = 8) and TEsL (n = 6). Median (and range) of RNA integrity numbers (RINs) were 7.9 (TNL; 7.2–8.3), 7.6 (TEaL; 6.7–7.9) and 8.0 (TEsL; 7.6–8.2). PCA visualisation of variance [35] at whole transcriptome level (Fig 1) showed TNL was the group with biological replicates that shared greatest similarity to each other. In contrast, biological replicates for TEaL broadly formed two clusters, whereby half mostly overlapped with TNL and the other half were more similar to TEsL samples; TEsL whole transcriptome profiles showed relatively less overlap with TNL than TEaL samples.

DESeq2, edgeR and baySeq analysis were combined to identify statistically significant DEGs, which were consistently identified as differentially expressed despite high variance between overall transcriptome profiles. DEGs with a *q* value (i.e. FDR-adjusted *p* value) ≤0.05 in at least two of these methods was defined as a ‘shared’ potential DEG. To remove background noise derived from genes with a low expression level but a high FC for each two-group comparison, these shared DEGs were filtered according to the following rationale: if their median TPM was <1 in any one of the two sample groups, they were only designated as a ‘robust’ DEG for further analysis if FC was still ≥1.5 when this median TPM value was artificially set to 1 [36]. Total numbers of DEGs, both before and after this FC≥1.5 filtering, are shown in Table 5 (full lists of filtered DEGs in S1 Dataset). TEsL *vs* TNL was associated with 5.9 (before FC≥1.5 filtering) and 7.6 (after FC≥1.5 filtering) fold more DEGs than TEaL *vs* TNL; thus, the further the progress of cervical dilatation, the more differential gene expression occurred in the myometrium at labour (albeit a small proportion of all genes that contribute to the total myometrial transcriptome).

**Table 5:** Number of differentially expressed genes in human myometrium identified by RNA-seq for different labour states.

Condition (T2 *vs* T1)		DESeq	edgeR	baySeq	Shared	After FC≥1.5 filtering	↓ T2 relative to T1	↑ T2 relative to T1
*Cervical Dilatation*	TEaL *vs*. TNL	6	189	128	60	33	3	30
	TEsL *vs*. TNL	842	403	53	354	251	58	193
	TEsL *vs*. TEaL	0	1	11	1	0	0	0
*Fetal Membrane Rupture*	TL-ROM *vs*. TNL	13	96	81	37	20	6	14
	TL+ROM *vs*. TNL	838	701	151	578	426	43	383
	TL+ROM *vs*. TL-ROM	2	5	5	0	0	0	0

Down-regulated (↓) and up-regulated (↑) genes during term (37^+3^–41^+1^ weeks) gestation singleton pregnancy/labour; labour status defined at time of Caesarean section. *Abbreviations*: T1, sample group 1; T2, sample group 2; FC, median fold change; ROM, fetal membrane rupture; TNL, no labour (no cervical dilatation and no ROM); TEaL, early labour (≤3 cm cervical dilatation); TEsL, established labour (>3 cm cervical dilatation); TL-ROM, labour with 0–1 h ROM; TL+ROM, labour with >1 h ROM.

### DEGs associated with labour classified by status of ROM

To assess the overall impact of ROM on myometrial transcriptomes, using the same analyses methods applied to labour grouped by cervical dilatation, the same RNA-seq cohort of samples from labouring women were grouped as TL-ROM (n = 8) and TL+ROM (n = 6) instead to generate a separate output of DEGs (Table 3). Median ROM duration was ~9 hours (~4–26 hours range) for TL+ROM, and only the artificial ROM case (sample #66; Fig 1) was >24 hours ROM prior to Caesarean section. Median cervical dilatation was 3 cm for both TL-ROM (1–8 cm range) and TL+ROM (2–6 cm range), which showed no statistically discernible difference (*p* = 0.87) unlike cervical dilatation status at TEaL (2 cm; 1–3 cm) *vs* TEsL (4 cm, 4–8 cm) where *p* = 0.0003. Median and range of RINs were 7.7 (TL-ROM; 6.7–8.2) and 7.8 (TL+ROM; 7.4–8.2).

PCA plot annotation for ROM status (Fig 1) showed TL-ROM samples were relatively less distinct than TL+ROM when compared to TNL. One TL+ROM sample (labelled ‘#51’ at Fig 1) showed no distinction in its overall transcriptomic profile from TNL samples despite being associated with a ROM duration of ~9 hours. DEGs identification and FC≥1.5 filtering for ROM groupings were undertaken as described for cervical dilatation groupings; total DEG numbers and full lists of filtered DEGs are provided in Table 5 and S2 Dataset, respectively. TL+ROM *vs* TNL had 15.6 (before FC≥1.5 filtering) and 21.3 (after FC≥1.5 filtering) fold more DEGs than TL-ROM *vs* TNL; thus, more myometrial genes were differentially expressed at labour when ROM was present than absent, and ROM was associated with more DEGs than cervical dilatation.

### Differences between labour classifications at enriched GO terms

GO enrichment was undertaken with consideration of both the number of DEGs assigned to each GO term and their expression FC values; terms for biological process (BP), molecular function (MF) and cellular component (CC) GO classes were identified. For labour grouped by cervical dilatation, GO terms enriched from TEaL *vs* TNL and TEsL *vs* TNL for all three classes are presented in S3 Dataset, and the top five BP terms are listed in Table 6. Immune/inflammation-related BP terms dominated the up-regulated DEGs at both TEaL and TEsL; whereas for down-regulated DEGs, ‘rhythmic process’ was the only enriched BP term for TEaL and the top five BP terms for TEsL were all related to the regulation of muscle contractions. For labour grouped by ROM status, all GO terms for BP, MF and CC classes enriched from TL-ROM *vs* TNL and TL+ROM *vs* TNL are presented in S4 Dataset, and the top five BP terms are also listed in Table 6. As with cervical dilatation groupings, immune/inflammation-related processes dominated the top five BP terms for up-regulated DEGs at both TL-ROM and TL+ROM, some of which were the same to those enriched by TEaL and TEsL. Contrastingly, no BP terms were enriched by DEGs down-regulated at TL-ROM, whereas TL+ROM down-regulated DEGs enriched ‘rhythmic process’ and ‘circadian rhythm’ terms.

**Table 6:** Top five enriched gene ontology (biological processes) terms for human myometrium in different labour states.

Comparison		Enriched GO terms (GO Identifier)	Raw *p* value	FDR-adjusted *p* value
*Cervical Dilatation*	TEaL **↑** *vs*. TNL	defense response (GO:0006952)	5.13 x 10^−12^	3.50 x 10^−9^
		complement activation (GO:0006956)	4.62 x 10^−11^	2.17 x 10^−8^
		phagocytosis (GO:0006909)	7.94 x 10^−11^	2.94 x 10^−8^
		inflammatory response (GO:0006954)	1.63 x 10^−10^	4.39 x 10^−8^
		immune response (GO:0006955)	4.16 x 10^−9^	5.21 x 10^−7^
	TEaL **↓** *vs*. TNL	rhythmic process (GO:0048511)	2.40 x 10^−4^	2.63 x 10^−2^
	TEsL **↑** *vs*. TNL	inflammatory response (GO:0006954)	1.48 x 10^−25^	6.97 x 10^−22^
		defense response (GO:0006952)	5.24 x 10^−19^	6.15 x 10^−16^
		immune response (GO:0006955)	2.18 x 10^−18^	2.05 x 10^−15^
		cell activation (GO:0001775)	8.90 x 10^−15^	5.23 x 10^−12^
		leukocyte activation (GO:0045321)	6.06 x 10^−13^	1.58 x 10^−10^
	TEsL **↓** *vs*. TNL	muscle contraction (GO:0006936)	1.78 x 10^−4^	2.75 x 10^−2^
		cardiac conduction (GO:0061337)	1.96 x 10^−4^	2.75 x 10^−2^
		action potential (GO:0001508)	2.65 x 10^−4^	3.01 x 10^−2^
		membrane repolarization (GO:0086009)	6.63 x 10^−4^	3.76 x 10^−2^
		membrane depolarization (GO:0051899)	1.19 x 10^−3^	4.84 x 10^−2^
*Fetal Membrane Rupture*	TL-ROM **↑** *vs*. TNL	phagocytosis, recognition (GO:0006910)	6.97 x 10^−12^	1.14 x 10^−9^
		phagocytosis, engulfment (GO:0006911)	1.02 x 10^−10^	9.97 x 10^−9^
		defense response (GO:0006952)	1.41 x 10^−6^	3.17 x 10^−5^
		endocytosis (GO:0006897)	3.49 x10^-6^	6.45 x 10^−5^
		membrane organization (GO:0061024)	4.85 x 10^−6^	8.81 x 10^−5^
	TL-ROM **↓** *vs*. TNL	N/A	N/A	N/A
	TL+ROM **↑** *vs*. TNL	inflammatory response (GO:0006954)	4.28 x 10^−33^	2.60 x 10^−29^
		defense response (GO:0006952)	4.10 x 10^−29^	1.25 x 10^−25^
		immune response (GO:0006955)	7.38 x 10^−28^	1.28 x 10^−24^
		cell activation (GO:0001775)	8.76 x 10^−22^	7.61 x 10^−19^
		leukocyte activation (GO:0045321)	1.34 x 10^−18^	5.82 x 10^−16^
	TL+ROM **↓** *vs*. TNL	rhythmic process (GO:0048511)	6.24 x 10^−6^	9.81 x 10^−3^
		circadian rhythm (GO:0007623)	1.98 x10^-4^	3.41 x 10^−2^

Down-regulated (↓) and up-regulated (↑) differential gene expression during term (37^+3^–41^+1^ weeks) gestation singleton pregnancy/labour; labour status defined at time of Caesarean section. *Abbreviations*: GO, gene ontology; FDR, false discovery rate; ROM, fetal membrane rupture; TNL, no labour (no cervical dilatation and no ROM); TEaL, early labour (≤3 cm cervical dilatation); TEsL, established labour (>3 cm cervical dilatation); TL-ROM, labour with 0–1 h ROM; TL+ROM, labour with >1 h ROM.

### Unique and shared DEGs across all labour classifications

**Fig 2:**
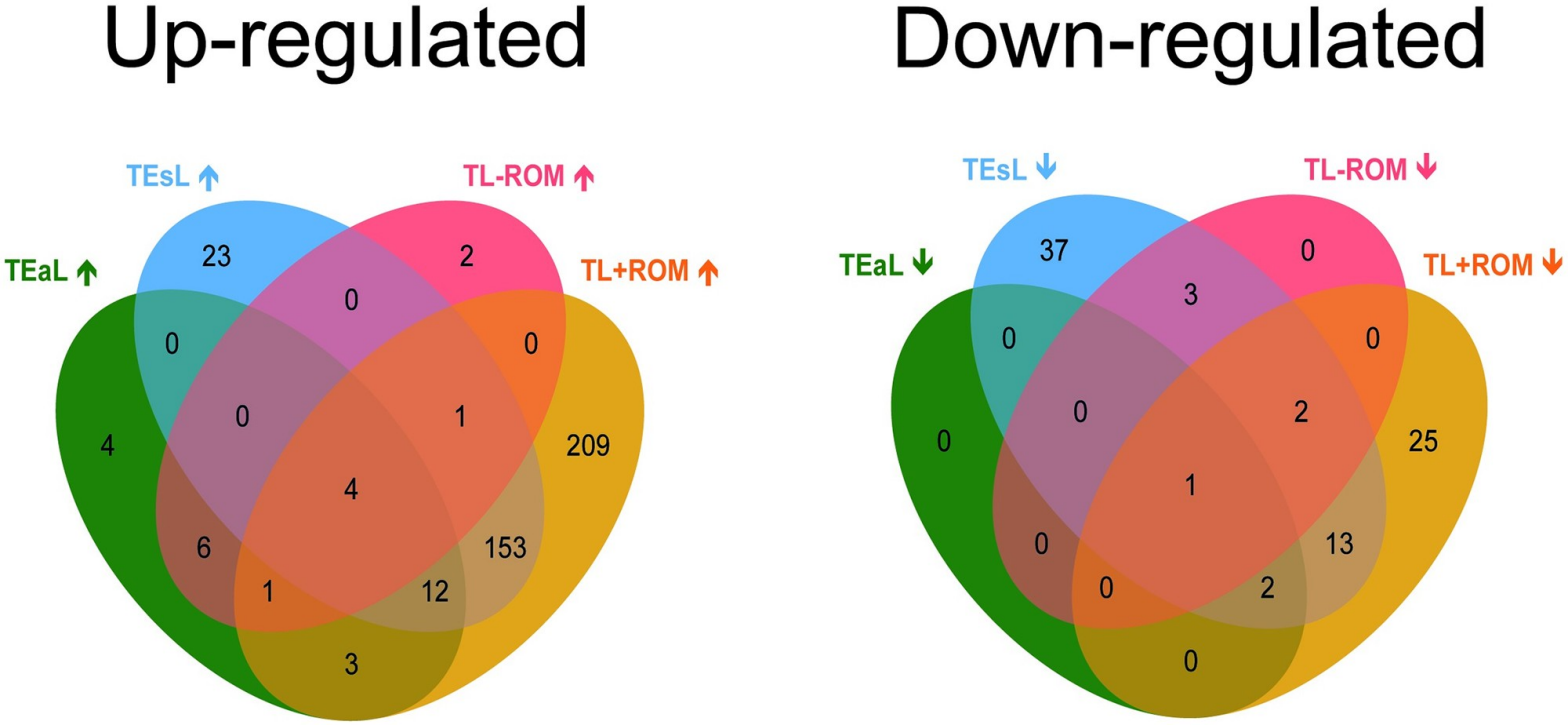
Grouping human myometrium FC≥1.5 DEGs according to cervical dilatation or ROM status at labour. Venn diagrams generated from lists of labour-associated up-regulated (↑) and down-regulated (↓) differentially expressed genes (DEGs) in myometrium biopsies, which were obtained from term gestation singleton pregnant women. Values shown only represent number of DEGs that demonstrated median fold change (FC) ≥1.5, when compared to the non-labouring state (TNL, n = 8), in transcript abundance for each of type of labour, which was classified by status of cervical dilatation or fetal membrane rupture (ROM; >1 hour prior to fetal delivery) at time of Caesarean section. For cervical dilatation, labouring women were grouped as either in early (≤3 cm; TEaL, n = 8) or established (>3 cm; TEsL, n = 6) labour. For ROM, women were grouped as either labouring in the absence (TL-ROM, n = 8) or presence (TL+ROM, n = 6) of ROM.

Venn diagrams (Fig 2) show the numbers of DEGs for TEaL, TEsL, TL-ROM and TL+ROM, each relative to TNL, that were unique or shared amongst all four labour groups. For up-regulated transcription, TL+ROM had the most and TL-ROM had the least unique DEGs. For down-regulated transcription, TEsL had the most unique DEGs and none were found for both TEaL and TL-ROM. Four up-regulated DEGs were shared between all labour classifications; namely *AREG*, *LIF*, *LILRA5* and *NAMPT* (S5 Dataset). Only one down-regulated DEG, *PER3*, was shared between the same labour groups (S6 Dataset). Median FC and *q* values from differential expression analysis for these five DEGs are listed in Table 7 (summarised from S1 and S2 Datasets).

**Table 7:** RNA-seq summary for differentially expressed genes common to four labour classifications in human myometrium.

Condition		Gene Symbol	Median Fold Change (high:low TPM)	Differential Expression Analysis *q* values		
				DESeq	EdgeR	baySeq
*Cervical Dilatation*	TEaL *vs*. TNL	*AREG*	2.97	n/a	3.55 x 10^−4^	2.18 x 10^−3^
		*LIF*	1.51	n/a	4.86 x 10^−4^	1.99 x 10^−4^
		*LILRA5*	2.01	n/a	5.21 x 10^−3^	2.69 x 10^−3^
		*NAMPT*	1.54	n/a	2.85 x 10^−3^	4.42 x 10^−3^
		*PER3*	4.06	1.77 x 10^−5^	4.30 x 10^−6^	3.98 x 10^−4^
	TEsL *vs*. TNL	*AREG*	2.68	2.22 x 10^−7^	2.17 x 10^−8^	2.29 x 10^−3^
		*LIF*	2.22	1.50 x 10^−3^	3.23 x 10^−6^	3.74 x 10^−4^
		*LILRA5*	3.11	4.52 x 10^−5^	3.52 x 10^−6^	5.07 x 10^−3^
		*NAMPT*	2.90	3.93 x 10^−5^	1.00 x 10^−6^	1.03 x 10^−2^
		*PER3*	2.42	4.34 x 10^−4^	1.43 x 10^−4^	4.85 x 10^−2^
*Fetal Membrane Rupture*	TL-ROM *vs*. TNL	*AREG*	2.68	4.24 x 10^−4^	1.70 x 10^−5^	2.83 x 10^−3^
		*LIF*	1.85	n/a	5.35 x 10^−3^	7.04 x 10^−3^
		*LILRA5*	2.23	n/a	1.14 x 10^−2^	1.56 x 10^−2^
		*NAMPT*	1.66	n/a	1.42 x 10^−2^	4.26 x 10^−2^
		*PER3*	2.84	3.83 x 10^−3^	2.63 x 10^−4^	4.69 x 10^−3^
	TL+ROM *vs*. TNL	*AREG*	3.36	3.32 x 10^−3^	3.28 x 10^−6^	2.00 x 10^−3^
		*LIF*	9.71	3.53 x 10^−4^	5.80 x 10^−8^	5.61 x 10^−5^
		*LILRA5*	3.11	1.26 x 10^−3^	1.04 x 10^−5^	1.30 x 10^−3^
		*NAMPT*	6.01	2.31 x 10^−4^	3.55 x 10^−7^	2.18 x 10^−3^
		*PER3*	2.90	3.36 x 10^−5^	4.11 x 10^−6^	4.01 x 10^−3^

GeneID in S1 and S2 Datasets: ENSG00000109321 (*AREG*), ENSG00000128342 (*LIF*), ENSG00000187116 (*LILRA5*), ENSG00000105835 (*NAMPT*), ENSG00000049246 (*PER3*). Values associated with increased (*AREG*, *LIF*, *LILRA5* and *NAMPT*) or decreased (*PER3*) expression at labour relative to the non-labouring state. Labour status defined at time of Caesarean section. *Abbreviations*: TPM, transcripts per million; ROM, fetal membrane rupture; TNL, no labour (no cervical dilatation and no ROM); TEaL, early labour (≤3 cm cervical dilatation); TEsL, established labour (>3 cm cervical dilatation); TL-ROM, labour with 0–1 h ROM; TL+ROM, labour with >1 h ROM.

### Myometrial DEGs of all labour classifications in placenta and choriodecidua

**Fig 3:**
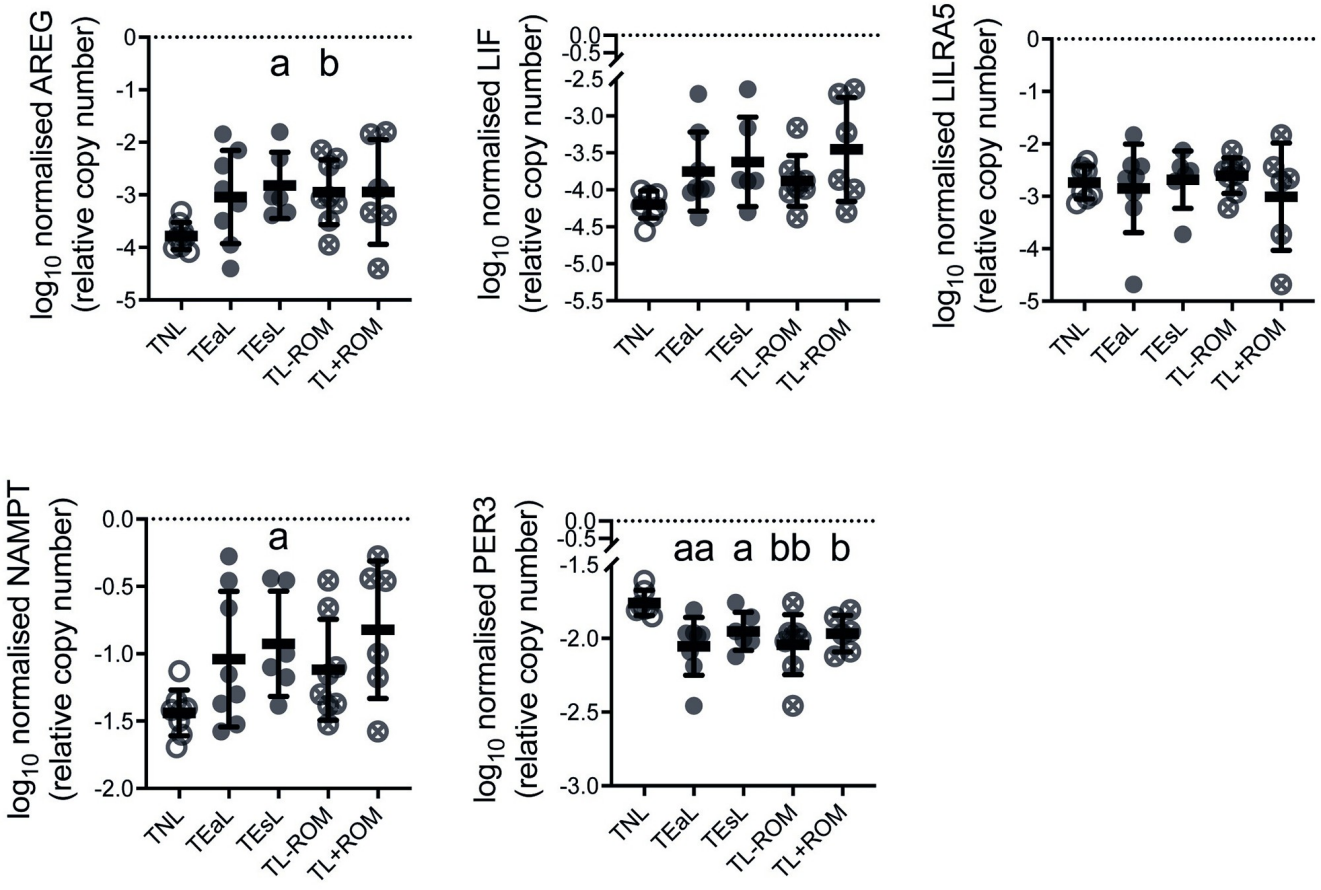
Relative mRNA abundance determined by qPCR for transcriptomics-identified myometrial DEGs common to four labour classifications in RNA-seq cohort tissues. Log_10_ transformed qPCR data (mean with standard deviation; n = 6–8) for myometrial mRNA levels of genes identified by RNA-seq (Fig 2) to be differentially expressed (DEGs) for all four classifications of labour, when compared to the non-labouring state (TNL), in the same biopsies. For cervical dilatation, labour was classified as early (≤3 cm; TEaL) or established (>3 cm; TEsL) at time of Caesarean section. These TEaL and TEsL samples were alternatively classified by fetal membrane rupture (ROM) status, whereby either ROM was absent (TL-ROM) or present (TL+ROM) for >1 hour prior to fetal delivery, to assess the effect of ROM irrespective of cervical dilatation. All data for DEGs of interest were normalised to both β2-microglobulin (*B2M*) and ribosomal protein L19 (*RPL19*). Brown-Forsythe & Welch ANOVA (Dunnett’s T3 *post-hoc*) was used for statistical analyses of TEaL / TEsL *vs* TNL (^a^ *p*≤0.05, ^aa^ p≤0.01) and TL-ROM / TL+ROM *vs* TNL (^b^ *p*≤0.05, ^bb^ p≤0.01).

**Fig 4:**
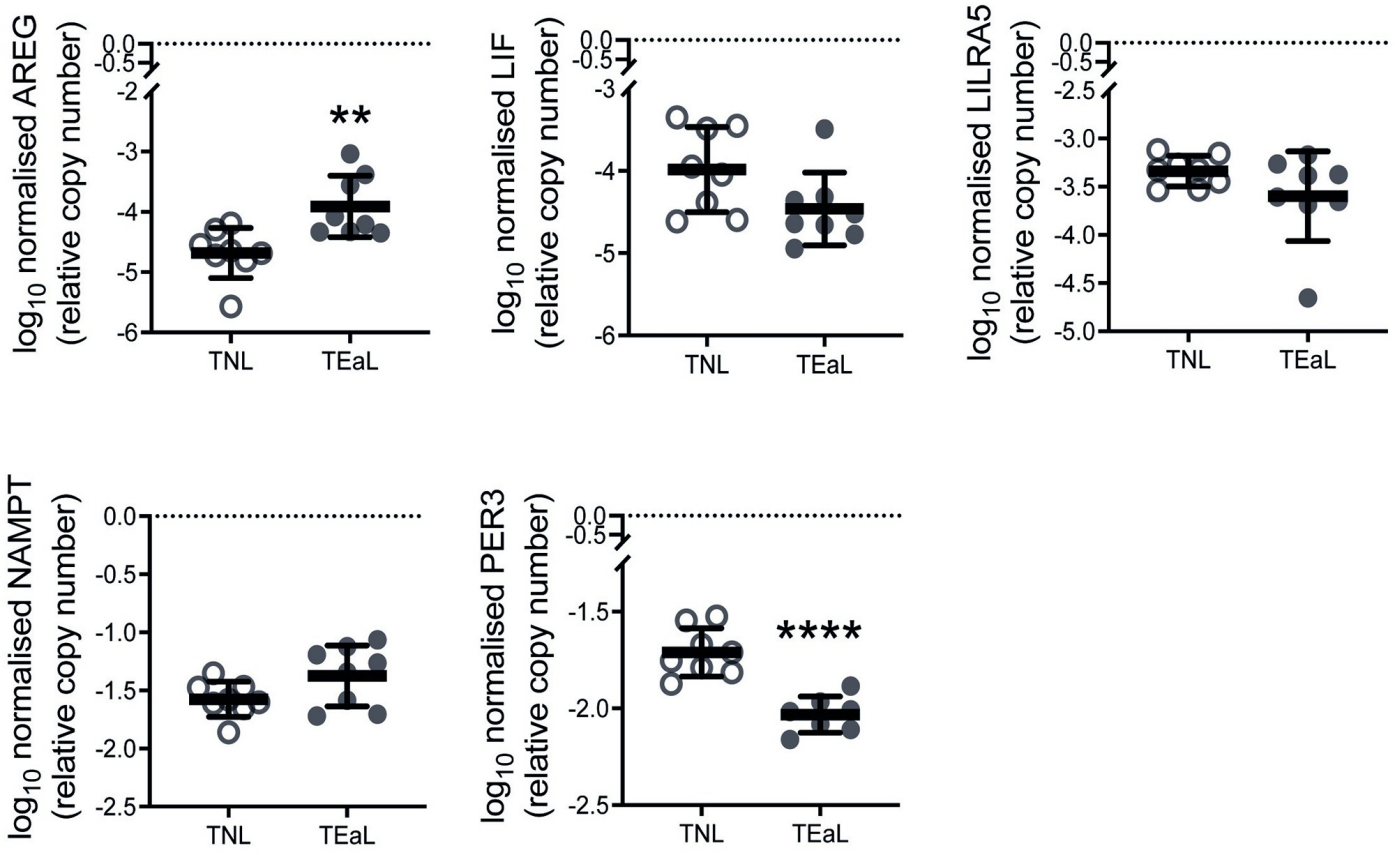
Relative mRNA abundance determined by qPCR for transcriptomics-identified myometrial DEGs common to TEaL in second cohort tissues. Log_10_ transformed qPCR data (mean with standard deviation; n = 7–8) for myometrial mRNA levels of genes identified by RNA-seq (Fig 2) to be differentially expressed (DEGs) during early phase labour (defined as ≤3 cm cervical dilatation; TEaL), when compared to the non-labouring state (TNL), in biopsies obtained from a cohort of term gestation singleton pregnant women separate to those in the RNA-seq cohort. All data for DEGs of interest were normalised to both β2-microglobulin (*B2M*) and ribosomal protein L19 (*RPL19*). Two-tailed Welch’s *t*-test was used for statistical analysis of TEaL *vs* TNL (** *p*≤0.01, **** *p*≤0.0001).

Myometrial *AREG*, *LIF*, *LILRA5*, *NAMPT* and *PER3* mRNA abundance was assessed by qPCR to further determine their status as common DEGs for all four classifications of labour (Fig 2) using RNA-seq samples and those from a second cohort of women; the latter were grouped as TNL (n = 8) and TEaL (n = 8, median 1.5 cm (1–3 cm range) cervical dilatation; comprised of n = 5 for TL-ROM and n = 3 for TL+ROM) (Table 4). For the RNA-seq cohort, labour-associated changes to all except *LILRA5* were consistent to what was observed from transcriptome analysis albeit to different extents of ANOVA-based significance (Fig 3). For the second cohort, where only the TEaL *vs* TNL comparison was considered, myometrial *AREG* and *PER3* expression patterns were the most consistent to those identified from RNA-seq samples (Fig 4). *PER3* and *NAMPT* are regulated by circadian rhythm [37] but differences in times of fetal delivery for each labour group *vs* TNL were not statistically significant for both cohorts (Tables 2–4; S1 Fig).

**Fig 5:**
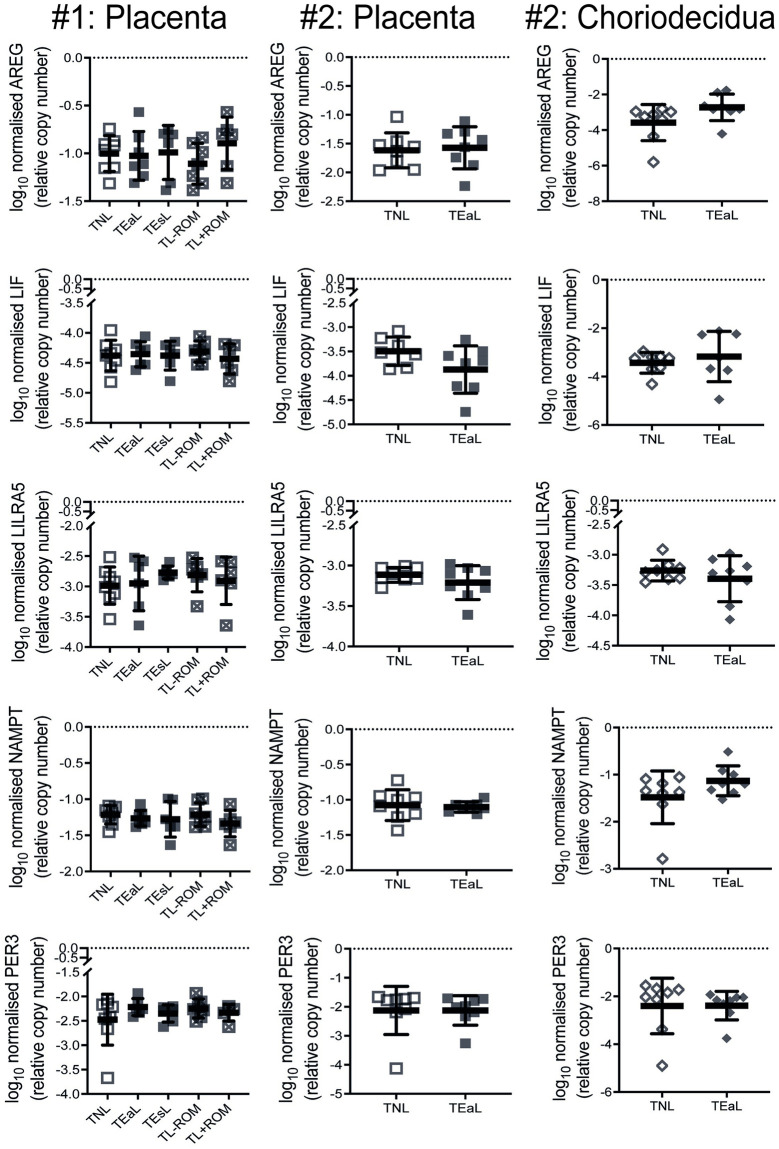
Placenta and choriodecidua tissue expression levels for transcriptomics-identified myometrial DEGs common to four labour classifications. Log_10_ transformed qPCR data (mean with standard deviation) for mRNA expression levels in placenta and choriodecidua biopsies of genes identified from myometrium RNA-seq data (Fig 2) to be differentially expressed (DEGs) for four classifications of labour, when compared to the non-labouring state (TNL), in biopsies from the RNA-seq (‘#1’; n = 5–8) and second (‘#2’; n = 7–8) cohorts of term gestation singleton pregnant women. For cervical dilatation, labour was classified as early (≤3 cm; TEaL) or established (>3 cm; TEsL) at time of Caesarean section. These TEaL and TEsL samples were alternatively classified by fetal membrane rupture (ROM) status, whereby either ROM was absent (TL-ROM) or present (TL+ROM) for >1 hour prior to fetal delivery, to assess the effect of ROM irrespective of cervical dilatation. Data for all DEGs of interest were normalised to both β2-microglobulin (*B2M*) and ribosomal protein L19 (*RPL19*). For statistical analyses of cohort #1 data, Brown-Forsythe & Welch ANOVA (Dunnett’s T3 *post-hoc*) was used for TEaL / TEsL *vs* TNL and TL-ROM / TL+ROM *vs* TNL; all p>0.05. For statistical analysis of cohort #2 data, Welch’s *t*-test was used for TEaL *vs* TNL; all p>0.05.

RNA extracts from placenta biopsies obtained from the same two cohorts of women, along with choriodecidua available for only the second cohort, were also analysed by qPCR for their expression of these five genes to determine whether they follow the same patterns as their patient-matched myometrium. From this, placental mRNA levels for these five genes were found to not be different for all labour groups relative to TNL (Fig 5); the same was observed for choriodecidual mRNA abundance at TEaL *vs* TNL comparison for the second sample cohort.

## Discussion

We used our RNA-seq dataset to show the extent to which transcriptomic analysis of myometrium biopsies from healthy term labouring women can produce different outcomes for different states of cervical dilatation and ROM. Thus, our present work demonstrates that this physiologically dynamic process [22] cannot be represented by a single state of the myometrial transcriptome from initiation to completion. To identify signalling networks specifically involved in ensuring that labour starts at the right time, which will be the most promising therapeutic targets for preventing its mistiming, it is important for samples of interest to represent the beginning (rather than the middle or end) of labour. This is an important consideration for use of transcriptomics, when it is common practice for individual (non-integrated) RNA-seq studies to comprise of 2–6 samples per group as a reasonable compromise between economic cost and variance minimisation [38, 39]; the latter is particularly difficult for human samples and makes stringent clinical phenotyping necessary [40, 41].

PCA, which was used to visualise overall (unfiltered) differences between transcriptome profiles, showed a high degree of variance for myometrium biopsies from labouring women. Relatively tighter clustering for TNL samples suggests that labour-associated variance was caused by biological, rather than technical, factors. Upon filtering at differential expression analysis, the absence of FC≥1.5 DEGs from comparison of labour groups to each other (i.e. TEsL *vs* TEaL and TL+ROM *vs* TL-ROM) emphasised the impact of variance specifically for samples from labouring women. It is likely that no FC≥1.5 DEGs were identified because neither labour group in each paired analysis contained sufficiently similar TPM values between biological replicates for FCs to be consistent. Similarities between labouring samples for status of ROM (at TEaL *vs* TEsL) or cervical dilatation (at TL+ROM *vs* TL-ROM) may have minimised differences at PCA. High variance may also be explained by human myometrial tissue heterogeneity, which arises from its physiological state, specifically distribution of functional output (i.e. contractile activity) at organ level, and/or cell type diversity during labour.

Functional heterogeneity at the myometrium has been evidenced by studies that measured *in vivo* labour contractions, which showed that they occur at discreet and randomly localised regions of the uterus, rather being evenly distributed in force and frequency across the entire organ, especially during its onset [42, 43]. Such unpredictable distribution of myometrial contractility, especially at the beginning of labour, makes it difficult to confirm whether the 60–100 mg tissue samples used for RNA-seq were all representative of uterine regions that were activated or quiescent to the same extent for each set of biological replicates [44]; their overall transcriptome differences may thus reflect their difference in activation status. Cell type heterogeneity also exists in the myometrium, which consists of mostly myometrial smooth muscle cells but also contains leukocytes, Cajal-like interstitial cells, fibroblasts and vascular smooth muscle cells [45–47]. Bulk RNA-seq is not designed to delineate the transcriptomes of different cell types. Thus, our findings support the use of single cell (sc)RNA-seq [48–50] and “computational deconvolution” [51, 52], ideally in an integrative manner [53], to determine whether it is tissue function and/or cell population heterogeneity that impacts on human myometrial transcriptome profiling the most at labour.

Myometrial DEGs identification showed labour mostly enhances (rather than suppresses) transcriptional activity and ROM is its biggest driver. GO analysis was only used for robust DEGs that were most consistent in their expression patterns despite apparent sample heterogeneity. Enrichment of immune/inflammation-related GO terms by up-regulated DEGs for all labour groups was expected because parturition is generally accepted as an inflammatory process [46, 54–56]. Cells of the immune system, along with those of the reproductive tissues, can release pro-inflammatory mediators [57, 58] that potentially increase the expression of myometrial contraction-associated genes [59]. However, other GO terms that were also enriched should not be ignored because immunology-related GO terms are the most well-annotated in knowledgebases and thus can be overrepresented by GO analyses [60, 61]. For down-regulated FC≥1.5 DEGs, their low frequency for all labour groups (relative to TNL) resulted in less, or none in the case of TL-ROM, enriched GO terms when compared to up-regulated DEGs. Technical bias caused by insufficient GO annotation within knowledgebases may have also resulted in lack of GO enrichment for down-regulated DEGs [62]. Afterall, there were more enriched GO terms for up-regulated TL-ROM than down-regulated TL+ROM DEGs, despite the former having 3.1-fold less FC≥1.5 DEGs.

Down-regulated FC≥1.5 DEGs did not enrich immune/inflammation-related GO terms. Instead, rhythmic process/circadian rhythm GO terms were enriched for TEaL and TL+ROM. Roles of circadian rhythm genes in labouring human myometrium have so far not been directly assessed but there is rationale from related research to support focus on their contribution. Rhesus macaques *in vivo* spontaneous uterine contractures follow a maternal circadian rhythm [63, 64]. A nocturnal peak in circulating oxytocin concentration has been observed in pregnant rhesus macaques [65] and women [66]. Chronodisruption during human pregnancy [67] has been associated with increased rates of adverse outcomes [68–71]. For TEsL, we observed a down-regulation of GO terms associated with muscle contractions. This would appear to suggest enhanced cervical dilatation coincides with reduced myometrial contractility, at least in the lower uterine segment where relaxation near the cervix may aid fetal expulsion. Alternatively, these transcriptomic changes may indicate negative feedback in response to sufficient accumulation of proteins required to maintain contractility for the rest of the labouring process. Proteomics and physiology-based comparisons of TEsL and TEaL will be needed to determine whether either interpretation is true.

Only *AREG*, *LIF*, *LILRA5*, *NAMPT* and *PER3* were identified as differentially expressed throughout labour. Additional analysis using qPCR helped to demonstrate whether these five DEGs are worthy of further investigation, especially for instances when high sensitivity techniques like RNA-seq are not readily available. *LILRA5* was not observed as a DEG using qPCR and, unlike the other four DEGs, is known only to be expressed in leukocytes and other hematopoietic cells [72]. Only *AREG* and *PER3* were identified by qPCR as myometrial DEGs for two different cohorts of women, which made them the most convincing DEGs for labour. Furthermore, labour-related *AREG* and *PER3* expression was specific to myometrium, at least when compared to *in utero* adjacent choriodecidua and placenta. Lack of differential expression in placenta, which is more vascularised than myometrium, suggests changes in myometrial *AREG* and *PER3* expression at labour is unlikely due to dominance of transcriptional activity by surrounding leukocytes.

In the end, only two myometrial DEGs were confidently identified to be relevant to onset at labour out of a possibility of 20465 protein-coding genes in the Ensembl knowledgebase; namely *AREG* and *PER3*. A key limitation of the present study was that cervical dilatation and ROM status groupings had to be analysed independently of each other (using the same pool of 15 samples) due to a small cohort. A larger sample cohort that would allow each TEaL and TEsL group to be sub-divided by ROM status (rather than each being a mix of TL-ROM and TL+ROM) could potentially reveal more distinct labour-related PCA clustering, as well as provide more certainty to the identification of DEGs associated specifically with the start of labour. Nevertheless, there is evidence from previous research that supports further assessment of *AREG* and *PER3* as novel labour-related DEGs, which may make their signalling pathways the most promising therapeutic targets for reducing the risk of labour mistiming. *AREG* encodes amphiregulin, a ligand of epidermal growth factor receptors (EGFRs), which are expressed in human uterine tissues at labour [73] and EGFR signalling was identified as relevant to human parturition by previous integrative analysis [21]. *PER3* is a circadian rhythm gene, which encodes a transcriptional repressor that controls the circadian clock system in peripheral tissues [74] and, incidentally, a genetic polymorphism within its ‘rs228669’ coding region [74] has been linked to high risk spontaneous preterm births [75].

## Conclusions

Refined sample groupings for our bulk RNA-seq dataset showed ROM, which typically occurs after uterine contractions have initiated, has substantial effects on the myometrial transcriptome. Thus, ROM, along with cervical dilatation, status needs to be defined when profiling myometrium biopsies for investigating mechanisms of labour onset. Our findings highlight the need to consider labour as a dynamic process that should not be represented by a single profile of changes at the uterus. Molecular events at different stages of labour need to be differentiated for its full characterisation from start to finish, which will increase the chance of discovering novel therapeutic targets with the highest potential in improving obstetric outcomes that are dependent on the timing of labour. Moving forward with the use of transcriptomics, alternative methodologies (with integrative approach) and additional sample cohorts are needed to determine whether our observation of low DEG numbers for the beginning of labour (represented mostly by TEaL and TL-ROM) was due to (i) cell type-specific localisation of labour-inducing transcriptomic changes, or (ii) changes at the proteome level or other aspect of myometrial activity playing a more vital role at labour onset than gene transcription. *AREG* and *PER3* were validated by qPCR out of the five DEGs shared between all four of our labour classifications, both of which are supported for further investigation in the context of labour onset by rationale from the findings of previous research; it will be interesting to see whether *AREG* or *PER3* remain as candidate DEGs after further studies.

## Supporting information

S1 Dataset Lists of up- and down-regulated FC≥1.5 DEGs for TEaL vs TNL and TEsL vs TNL comparisons. (XLSX)

S2 Dataset Lists of up- and down-regulated FC≥1.5 DEGs for TL-ROM vs TNL and TL+ROM vs TNL comparisons. (XLSX)

S3 Dataset Lists of enriched GO terms for up- and down-regulated FC≥1.5 DEGs at TEaL vs TNL and TEsL vs TNL comparisons. (XLSX)

S4 Dataset Lists of enriched GO terms for up- and down-regulated FC≥1.5 DEGs at TL-ROM vs TNL and TL+ROM vs TNL comparisons. (XLSX)

S5 Dataset Lists of shared and unique up-regulated FC≥1.5 DEGs (relative to TNL) for all labour classifications of interest. (XLSX)

S6 Dataset Lists of shared and unique down-regulated FC≥1.5 DEGs (relative to TNL) for all labour classifications of interest. (XLSX)

S1 Fig Times of birth for Caesarean section deliveries associated with biopsies from RNA-seq & second cohorts. Fetal delivery times for both cohorts of women from whom biopsies were obtained at term gestation singleton pregnancy for RNA-seq and/or qPCR; frequency of fetal deliveries pooled into 2-hour intervals are shown for a full day (24-hour) period. For the RNA-seq cohort, samples were obtained from non-labouring (TNL, n = 8), early labouring (≤3 cm cervical dilatation; TEaL, n = 8) or established labouring (>3 cm cervical dilatation; TEsL, n = 6) women; these TEaL and TEsL samples were alternatively grouped according to whether labour occurred in the absence (TL-ROM, n = 8) or presence (TL+ROM, n = 6) of fetal membrane rupture for >1 h prior to fetal delivery. Only TNL and TEaL groupings were used for samples from the second cohort of women. Statistical analysis was undertaken using Kruskal-Wallis (Dunn’s post-hoc; RNA-seq cohort) or Mann-Whitney (second cohort) tests to compare each labour group to their cohort-matched TNL group; all p>0.05.
(PDF)
