# Supplementary material for: Labour classified by cervical dilatation & fetal membrane rupture demonstrates differential impact on RNA-seq data for human myometrium tissues
Source: PLoS One. 2021 Nov 19;16(11):e0260119. doi: 10.1371/journal.pone.0260119 (PMC8604334; doi:10.1371/journal.pone.0260119)
Supplement: S1 Fig — Fetal delivery times for both cohorts of women from whom biopsies were obtained at term gestation singleton pregnancy for RNA-seq and/or qPCR; frequency of fetal deliveries pooled into 2-hour intervals are shown for a full day (24-hour) period. For the RNA-seq cohort, samples were obtained from non-labouring (TNL, n = 8), early labouring (≤3 cm cervical dilatation; TEaL, n = 8) or established labouring (>3 cm cervical dilatation; TEsL, n = 6) women; these TEaL and TEsL samples were alternatively grouped according to whether labour occurred in the absence (TL-ROM, n = 8) or presence (TL+ROM, n = 6) of fetal membrane rupture for >1 h prior to fetal delivery. Only TNL and TEaL groupings were used for samples from the second cohort of women. Statistical analysis was undertaken using Kruskal-Wallis (Dunn’s post-hoc; RNA-seq cohort) or Mann-Whitney (second cohort) tests to compare each labour group to their cohort-matched TNL group; all p>0.05. (PDF) [file pone.0260119.s007.pdf]

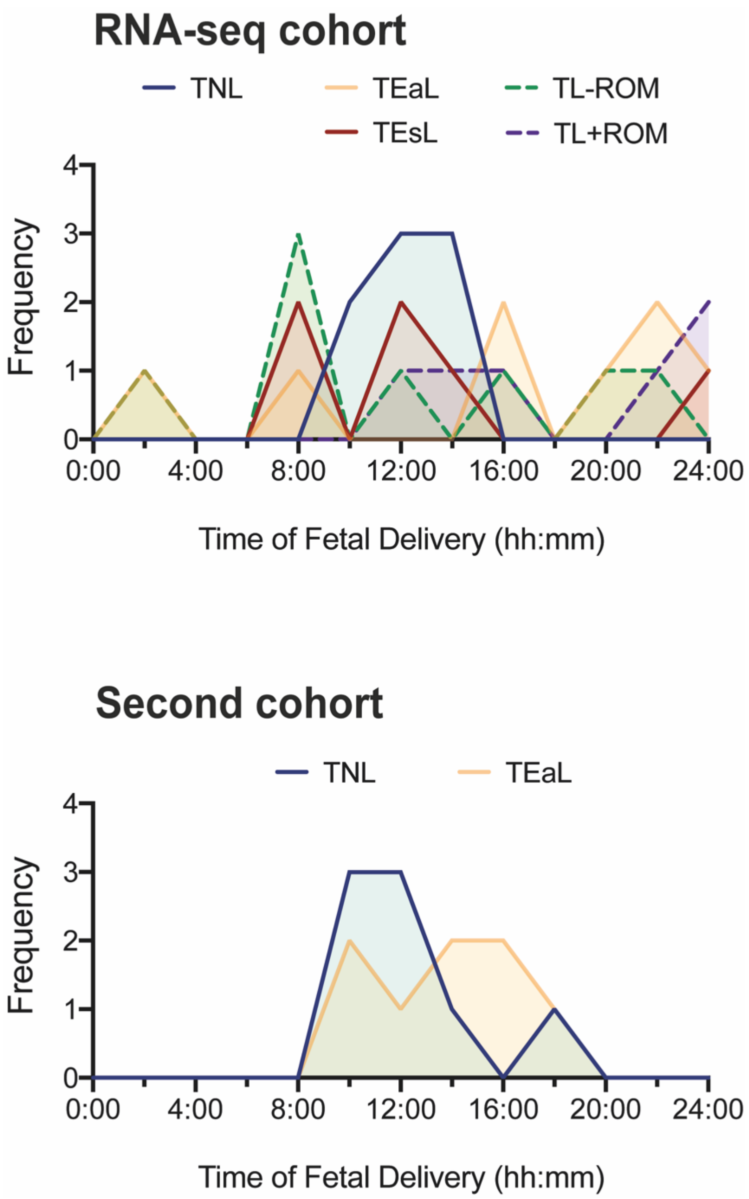

Fig S1. Times of birth for Caesarean section deliveries associated with biopsies from RNA-seq & second cohorts. Fetal delivery times for both cohorts of women from whom biopsies were obtained at term gestation singleton pregnancy for RNA-seq and/or qPCR; frequency of fetal deliveries pooled into 2-hour intervals are shown for a full day (24-hour) period. For the RNA-seq cohort, samples were obtained from non-labouring (TNL, n = 8), early labouring (≤3 cm cervical dilatation; TEaL, n = 8) or established labouring (>3 cm cervical dilatation; TEsL, n = 6) women; these TEaL and TEsL samples were alternatively grouped according to whether labour occurred in the absence (TL-ROM, n = 8) or presence (TL+ROM, n = 6) of fetal membrane rupture for >1 h prior to fetal delivery. Only TNL and TEaL groupings were used for samples from the second cohort of women. Statistical analysis was undertaken using Kruskal-Wallis (Dunn's *post-hoc*; RNA-seq cohort) or Mann-Whitney (second cohort) tests to compare each labour group to their cohort-matched TNL group; all $p>0.05$.
